# Supplementary material for: Improved state-level influenza nowcasting in the United States leveraging Internet-based data and network approaches
Source: Nat Commun. 2019 Jan 11;10:147. doi: 10.1038/s41467-018-08082-0 (PMC6329822; doi:10.1038/s41467-018-08082-0)
Supplement: Supplementary file 1 — Supplementary Information [file 41467_2018_8082_MOESM1_ESM.pdf]

Improved state-level influenza nowcasting in the United States leveraging Internet-based data and network approaches

Supplementary information

Lu et al.

Supplementary Methods

ARGO formulation

Let $y_{i,t}$ be the CDC %ILI in state i at time t, and let $\mathbf{X}_{i,t} = \{X_{i,t,k}\}_{k\in\{1,\ldots,M\}}$ be the vector of Internet-based data in corresponding state and time. ARGO assumes a hidden Markov model based on an autoregressive structure with N lags, as shown below:

$$\begin{array}{ccccccc} y_{i,1:N} & \longrightarrow & y_{i,2:(N+1)} & \longrightarrow & \cdots & \longrightarrow & y_{i,(T-N+1):T} \\ \downarrow & & \downarrow & & & & \downarrow \\ \mathbf{X}_{i,N} & & \mathbf{X}_{i,N+1} & & & & \mathbf{X}_{i,T} \end{array}$$

Here, the vectors $\{y_{i,(t-N+1):t}\}_{t\geq N}$ follow the Markov property, and at time T, the Tth such vector has not yet been fully observed. Meanwhile, the observed variables $\mathbf{X}_{i,T}$ depend only the corresponding hidden $y_{i,T}$.

ARGO model

The above formulation results in the model

$$y_{i,t} = \mu_i + \sum_{j=1}^{N} \alpha_j y_{i,t-j} + \sum_{k=1}^{M} \beta_k \mathbf{X}_{i,t,k} + \epsilon_t, \quad \epsilon_t \sim \mathcal{N}(0,\sigma^2) \tag{1}$$

We take $N = 52$ to incorporate short-term and seasonal autoregressive trends within the past year of data, and $M = 285$ at maximum, corresponding to the number of Google Trends and athenahealth variables. The high number of input variables gives us a $p > n$ situation, so we impose L_1 regularization. Therefore, we can solve for parameters μ_i, $\alpha = (\alpha_1, \ldots, \alpha_N)$, and $\beta = (\beta_1, \ldots, \beta_M)$ which minimize the objective function

$$\sum_t \left(y_{i,t} - \mu_i - \sum_{j=1}^{N} \alpha_j y_{i,t-j} - \sum_{k=1}^{M} \beta_k \mathbf{X}_{i,t,k} \right)^2 + \lambda_\alpha ||\alpha_j||_1 + \lambda_\beta ||\beta_k||_1 \tag{2}$$

using a rolling training window consisting of the 104 weeks prior to time t, with hyperparameters λ_α and λ_β.

Hyperparameters

The parameters in (2) are governed by hyperparameters λ_α and λ_β; however, we introduced a modification to the model. Rather than adhering to the groups α and β, we replaced them with more flexible groups in the following manner:

Let $\gamma = \{\alpha_1, \ldots, \alpha_N, \beta_1, \ldots, \beta_M\}$ be the set of regularized parameters. Take $p \subseteq \gamma$ to be a set of "priority" parameters, and $q = \gamma - p$ to be the remaining parameters. Letting γ_p and γ_q represent the parameter vectors corresponding to these sets, the objective function simply becomes

$$\sum_t \left(y_{i,t} - \mu_i - \sum_{j=1}^{N} \alpha_j y_{i,t-j} - \sum_{k=1}^{M} \beta_k \mathbf{X}_{i,t,k} \right)^2 + \lambda_p ||\gamma_p||_1 + \lambda_q ||\gamma_q||_1 \tag{3}$$

To limit the model space, we allowed p to take one of 3 configurations and selected the one with best out-of-sample performance within each state. The configurations are the parameters corresponding to:

1. athenahealth variables
2. athenahealth variables and the most correlated autoregressive terms, e.g. $\{y_{t-i}\}_{i \in \{1,2,3,6,12,52\}}$
3. athenahealth variables, the 2 most correlated Google Trends variables, and the most correlated autoregressive terms.

To further constrain the search space, we maintain a hyperparameter ratio $\lambda_p/\lambda_q = 1/10$. Since λ_p is smaller, this allows the priority parameters to take larger values, effectively giving them more weight in the regression. The single hyperparameter was then determined using 10-fold cross-validation over the training set. In practice, prediction accuracy was robust to the specific value of the ratio.

Net formulation

While the ARGO model for any given state i is constrained to the data within state i, we hypothesized that $y_{i,t}$ shows spatio-temporal structure with other states $s \neq i$ in the short term (i.e. over the past 4 weeks). Adopting the previous notation, the Net model is then

$$y_{i,t} = \mu_i + \sum_{j=1}^{52} \alpha_j y_{i,t-j} + \sum_{s \neq i} \sum_{k=0}^{3} \beta_{s,k} y_{s,t-k} + \epsilon_t, \quad \epsilon_t \sim \mathcal{N}(0, \sigma^2) \tag{4}$$

Like ARGO, this model was fit using a rolling 104-week window with 10-fold cross-validation for the regularization hyperparameter. However, for prediction at a given time t, the concurrent %ILI values $y_{s,t}$ are not yet observed, so we replace them with the corresponding real-time ARGO estimates $\hat{y}_{s,t}$. This substitution inherently assumes that ARGO is unbiased, i.e. $y_{i,t} = \hat{y}_{i,t} + \epsilon_t, \quad \epsilon_t \sim \mathcal{N}(0, \sigma^2)$.

Supplementary Figures

Fig 1: Time series plots displaying the performance of ARGO (blue) and ARGONet (red) over the period September 28, 2014 to May 14, 2017. The official CDC %ILI time series is shown in black. The AR52 benchmark is also displayed (gray dashes). The heatmap under each time series plot indicates which input model the ARGONet ensemble selected as its prediction each week.

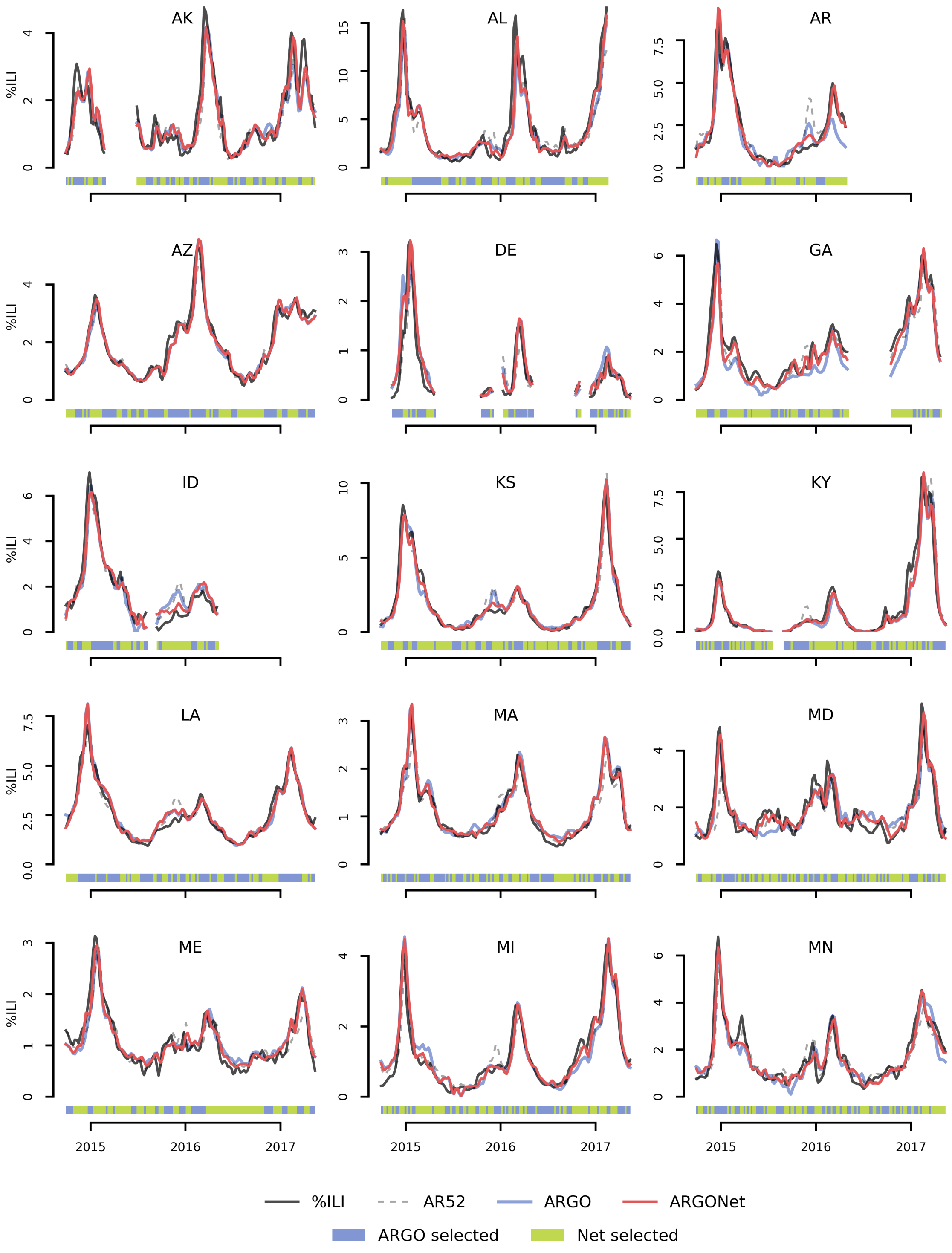

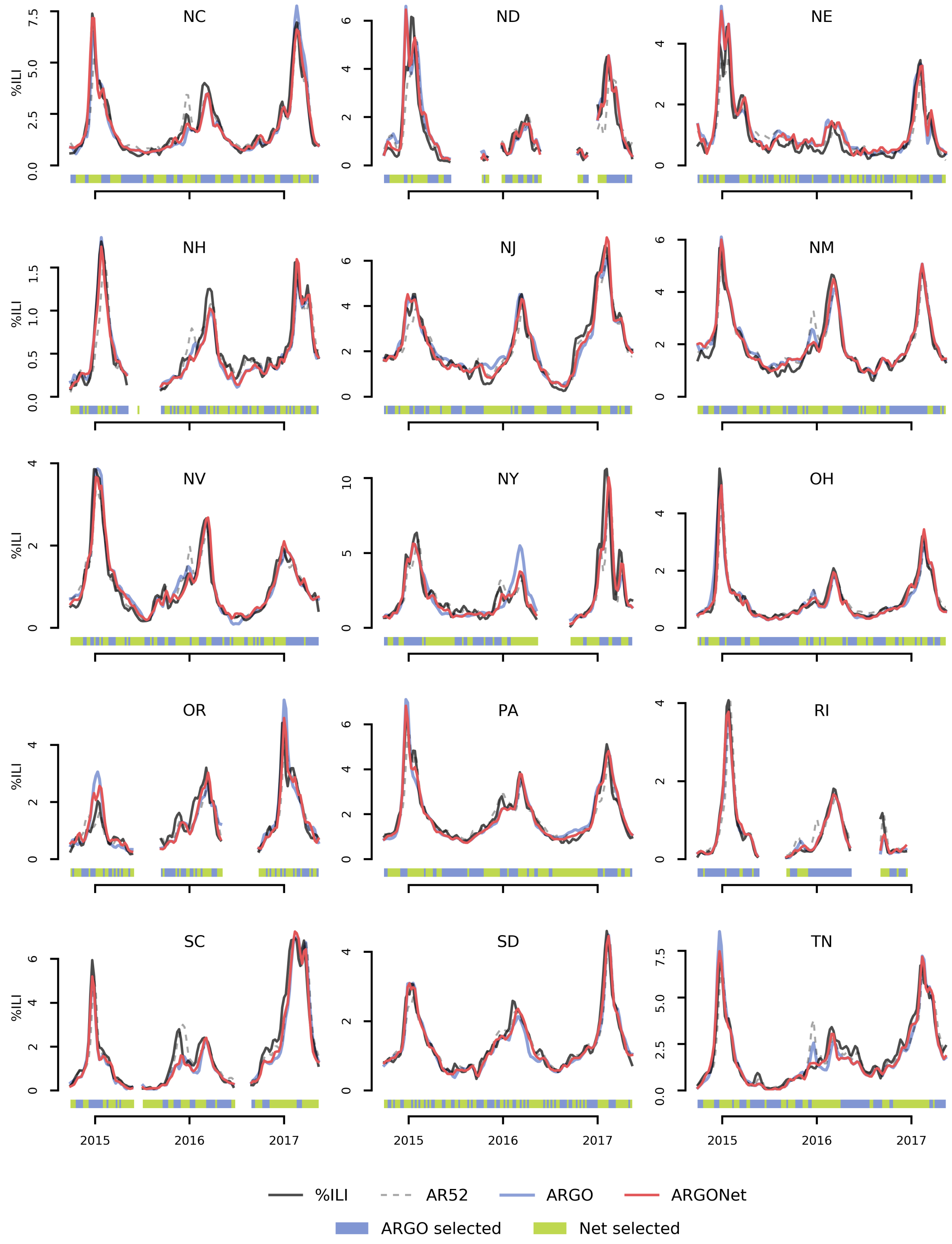
NC
ND
NE
NH
NJ
NM
NV
NY
OH
OR
PA
RI
SC
SD
TN
%ILI
2015
2016
2017
%ILI
AR52
ARGO
ARGONet
ARGO selected
Net selected

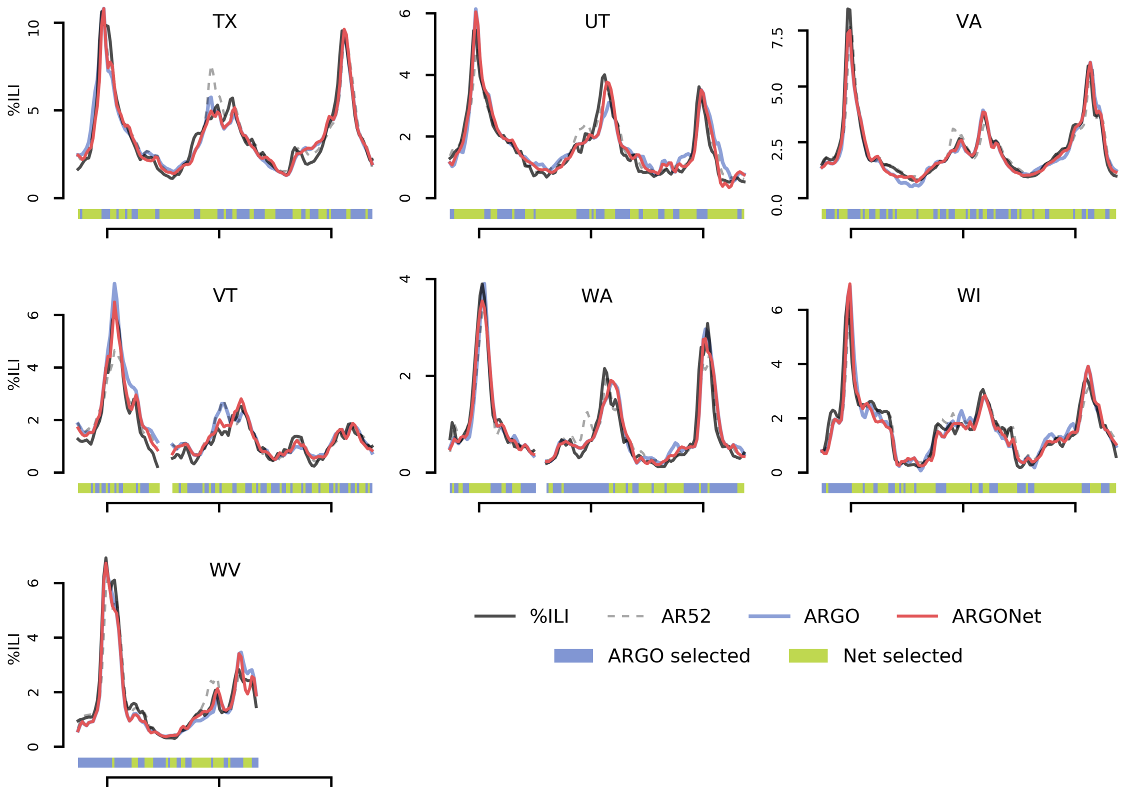

TX
UT
VA
VT
WA
WI
WV
%ILI
%ILI
AR52
ARGO
ARGONet
ARGO selected
Net selected

Fig 2: Factors associated with the performance improvement of our ensemble over the AR52 benchmark. a) Geographical heatmap of the improvement (%RMSE reduction) of ARGONet over AR52. Possible explanatory factors are b) population, which can affect data quality or spatial structure, c) the number of detectable flu-related Google Trends terms per state, which can be taken as a proxy of search data quality, and d) athenahealth coverage per state, calculated as average visits per thousand. e-i) Regression analysis indicates the presence of associations between e) ARGONet improvement and athenahealth coverage, with one outlier (Wisconsin, colored red) removed, f) ARGONet improvement and Google Trends quality, g) state population and Google Trends quality, h) ARGONet improvement and ILI curve deviation (computed as $\sigma(Y)/\mathrm{E}[Y]$), and i) ARGONet improvement and average healthcare providers reporting to CDC over the prediction period.

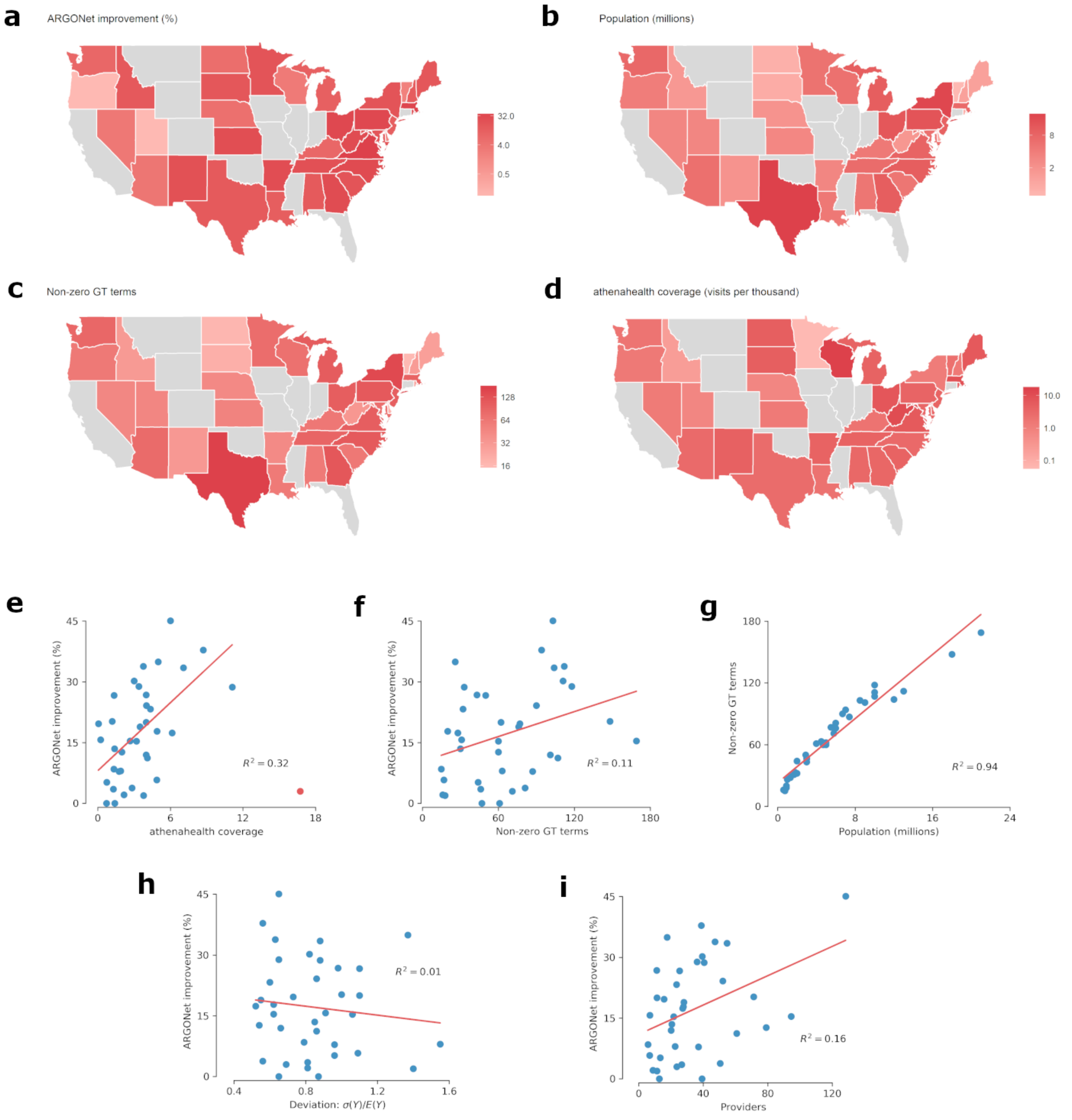

Supplementary Tables

Table 1: Aggregate metrics over each flu season for each state corresponding to the violin plots in Figures 1c and 3c. The reported numbers are the median and interquartile ranges of the aggregates. The final two rows are the best reported aggregates for each metric from Kandula et al. for comparison. These were not discussed in the main paper because of the different time period.

Model	RMSE	Correlation	MAPE
2014-15 to 2016-17 seasons			
ARGONet	0.48 (0.33-0.67)	0.90 (0.81-0.94)	0.22 (0.12-0.36)
Net	0.51 (0.37-0.71)	0.90 (0.80-0.93)	0.23 (0.17-0.39)
ARGO	0.51 (0.34-0.75)	0.89 (0.82-0.94)	0.24 (0.18-0.38)
AR52	0.60 (0.39-0.85)	0.83 (0.72-0.88)	0.25 (0.18-0.42)
2012-13 to 2014-15 seasons			
ARGO	0.56 (0.36-0.77)	0.93 (0.88-0.95)	0.25 (0.18-0.38)
AR52	0.65 (0.46-0.84)	0.87 (0.81-0.90)	0.24 (0.18-0.40)
GFT	0.98 (0.55-1.80)	0.91 (0.84-0.94)	0.47 (0.29-0.96)
2005-06 to 2010-11 seasons			
GFT	0.93 (0.66-1.33)	0.89 (0.80-0.94)	0.71 (0.44-1.51)
Kandula et al.	0.84 (0.54-1.25)	0.86 (0.75-0.91)	0.54 (0.33-0.90)

Table 2: Distribution of hyperparameter K for ARGONet model

K	States
1	AR, ID, MD, MI, MN, NE, NH, NV, OR, SD, VA, WI
2	AK, GA, KS, LA, ND, NJ, OH, RI, TX, UT, VT
3	AL, AZ, DE, KY, MA, ME, NC, NM, NY, PA, SC, TN, WA, WV

Table 3: Complete search queries downloaded from Google Trends

a influenza	acute bronchitis	anas barbariae hepatis	b flu
baby has rsv	baby rsv	baby with rsv	biaxin side effects
body temperature	braun thermoscan	break a fever	breaking a fever
bronchitis	can adults get rsv	can dogs get the flu from humans	chest cold
clarithromycin	cold	cold and flu	cold or flu
cold symptoms	cold versus flu	cold vs flu	cold with fever
contagious flu	cough and fever	cough fever	cough headache
coughing remedies	cure flu	cure the flu	dangerous fever
do i have the flu	duration of the flu	ear thermometer	early flu symptoms
expectorant	exposed to flu	exposure to flu	fever and cold
fever and cough	fever cough	fever flu	fever reducer
fight the flu	flu a	flu a and b	flu a symptoms
flu and bronchitis	flu and cold	flu and fever	flu and strep
flu care	flu children	flu cold	flu complications
flu contagious	flu contagious period	flu cough	flu cures
flu duration	flu fever	flu food	flu germs
flu gestation	flu headache	flu how long	flu in children
flu incubation	flu incubation period	flu lasts	flu length
flu like symptoms	flu medicine	flu or cold	flu or pneumonia
flu or strep	flu prophylaxis	flu recovery	flu recovery time
flu relief	flu remedies	flu remedy	flu report
flu reports	flu shot	flu shot symptoms	flu shots
flu symptoms	flu symptoms in toddlers	flu test	flu treatment
flu treatments	flu type	flu type a	flu type a symptoms
flu type b	flu type b symptoms	flu vaccine	flu versus cold
flu virus	flu vs cold	flu while pregnant	generic tamiflu
get over the flu	get over the flu fast	get rid of flu	get rid of the flu
get rid of the flu fast	getting over the flu	gripe	having the flu
high fever	home remedies for flu	how contagious is the flu	how long am i contagious
how long am i contagious with the flu	how long are you contagious	how long are you contagious with the flu	how long contagious
how long does flu last	how long does influenza last	how long does it take to get over the flu	how long does rsv last
how long does the flu last	how long does the flu last in adults	how long does the flu last?	how long flu
how long is flu contagious	how long is rsv contagious	how long is the flu	how long is the flu contagious
how to break a fever	how to break a fever in adults	how to cure the flu	how to get over flu
how to get over the flu	how to get rid of flu	how to get rid of the flu	how to reduce fever
how to treat flu	how to treat flu symptoms	how to treat the flu	human temperature
i have the flu	incubation for flu	incubation period flu	incubation period for flu
incubation period for influenza	incubation period for the flu	influenza a	influenza a and b
influenza a contagious	influenza a incubation	influenza a incubation period	influenza a symptoms
influenza a treatment	influenza b	influenza b symptoms	influenza b treatment
influenza contagious	influenza incubation	influenza incubation period	influenza symptoms
influenza treatment	influenza treatment guidelines	influenza type a	influenza type b
intestinal flu	is flu contagious	is influenza a contagious	is influenza contagious
is rsv contagious	is tamiflu an antibiotic	is the flu contagious	is the flu contagious?
medicine for flu	medicine for the flu	normal body	normal body temperature
oscillo	oscillococcinum	oseltamivir	over the counter flu
over the counter flu medicine	painful cough	pneumonia	positive flu test
pregnant and have the flu	rapid flu	recovering from flu	reduce a fever
reduce fever	remedies for flu	remedies for the flu	respiratory flu
robitussin	robitussin cf	robitussin cough	rsv
rsv baby	rsv contagious	rsv in adults	rsv infant
rsv infection	rsv symptoms	rsv symptoms in adults	rsv treatment
signs of flu	signs of rsv	signs of the flu	sinus
sinus infection cure	sinus infections	stomach flu	stomach flu symptoms
strep	strep throat	symptoms of bronchitis	symptoms of flu
symptoms of influenza	symptoms of influenza b	symptoms of pneumonia	symptoms of rsv
symptoms of rsv in adults	symptoms of the flu	taking temperature	tamiflu and alcohol
tamiflu and breastfeeding	tamiflu and pregnancy	tamiflu children	tamiflu contagious
tamiflu dosage	tamiflu dose	tamiflu drug	tamiflu drug interactions
tamiflu during pregnancy	tamiflu generic	tamiflu in children	tamiflu in pregnancy
tamiflu pediatric dosing	tamiflu side effects	tamiflu suspension	tamiflu while pregnant
tamiflu wiki	tessalon	the flu	the flu symptoms
the flu virus	treat flu	treat flu symptoms	treat the flu
treating flu	treating the flu	treatment for flu	treatment for rsv

treatment for the flu	tussin	tussionex	tylenol sinus
type a and b flu	type a flu	type a flu symptoms	type a influenza
type b flu	type b flu symptoms	type b influenza	upper respiratory
walking pneumonia	what is flu a	what is influenza	what is influenza a
what is influenza b	what is rsv	what is type a flu	what to do if you have the flu
what to eat when you have the flu	when is the flu contagious	when is the flu no longer contagious	when you have the flu
z pack and alcohol	thermoscan		

Table 4: (starting next page) Performance by each model over each season and state.

Metric	State	Model	2012-13	2013-14	2014-15	2015-16	2016-17	Whole Period	GFT Period	ARGONet Period
RMSE	AK	GFT	1.446	0.499	0.605	–	–	–	0.875	–
		AR52	0.611	0.74	0.61	1.034	0.825	0.717	0.622	0.78
		ARGO	0.587	0.65	0.663	0.844	0.784	0.663	0.605	0.727
		Net	0.782	0.766	0.767	0.88	0.721	0.709	0.701	0.728
		ARGONet	–	–	0.671	0.832	0.776	–	–	0.714
	AL	GFT	10.042	4.097	3.561	–	–	–	5.484	–
		AR52	1.379	0.653	2.594	3.307	3.04	2.026	1.448	2.608
		ARGO	1.368	0.574	2.09	3.803	2.384	1.96	1.241	2.522
		Net	1.2	0.587	1.629	3.215	1.839	1.649	1.021	2.108
		ARGONet	–	–	1.584	3.486	1.854	–	–	2.208
	AR	GFT	7.038	0.937	0.873	–	–	–	3.454	–
		AR52	0.656	0.989	1.382	1.136	–	0.926	0.894	1.125
		ARGO	1.368	1.011	1.062	1.13	–	1.007	0.997	0.991
		Net	0.884	0.817	1.168	0.585	–	0.774	0.827	0.829
		ARGONet	–	–	1.151	0.584	–	–	–	0.824
	AZ	GFT	0.999	0.396	0.524	–	–	–	0.567	–
		AR52	0.502	0.258	0.258	0.509	0.367	0.353	0.302	0.369
		ARGO	0.346	0.238	0.248	0.482	0.37	0.313	0.239	0.355
		Net	0.428	0.215	0.221	0.569	0.392	0.344	0.255	0.387
		ARGONet	–	–	0.229	0.487	0.373	–	–	0.355
	DE	GFT	0.709	1.186	0.733	–	–	–	0.883	–
		AR52	0.665	0.667	0.602	0.352	0.186	0.518	0.629	0.412
		ARGO	0.589	0.646	0.637	0.251	0.196	0.492	0.608	0.405
		Net	0.497	0.646	0.553	0.367	0.304	0.479	0.554	0.418
		ARGONet	–	–	0.614	0.271	0.23	–	–	0.404
	GA	GFT	5.371	1.413	1.784	–	–	–	2.763	–
		AR52	0.429	0.532	1.048	0.454	0.806	0.607	0.597	0.748
		ARGO	0.569	0.309	0.781	0.61	0.76	0.566	0.512	0.68
		Net	0.328	0.46	1.037	0.405	0.64	0.552	0.569	0.687
		ARGONet	–	–	0.639	0.473	0.589	–	–	0.532
	ID	GFT	3.833	0.932	0.793	–	–	–	2.083	–
		AR52	0.742	1.176	0.87	0.65	–	0.818	0.868	0.728
		ARGO	0.715	0.984	0.767	0.656	–	0.742	0.771	0.692
		Net	0.961	0.995	0.806	0.429	–	0.772	0.845	0.633
		ARGONet	–	–	0.703	0.453	–	–	–	0.614
	KS	GFT	2.558	0.582	0.957	–	–	–	1.322	–
		AR52	0.835	0.78	0.865	0.646	1.153	0.728	0.682	0.781
		ARGO	0.691	0.755	0.848	0.498	0.759	0.611	0.642	0.623
		Net	0.775	0.643	0.825	0.324	0.979	0.622	0.621	0.656
		ARGONet	–	–	0.779	0.343	0.769	–	–	0.573
	KY	GFT	1.449	0.311	0.479	–	–	–	0.757	–
		AR52	0.225	0.221	0.374	0.394	1.28	0.549	0.241	0.7
		ARGO	0.214	0.138	0.316	0.398	1.207	0.516	0.201	0.663
		Net	0.334	0.136	0.359	0.283	1.252	0.536	0.252	0.677
		ARGONet	–	–	0.325	0.276	1.197	–	–	0.644
	LA	GFT	4.331	0.699	0.477	–	–	–	2.074	–
		AR52	0.762	0.939	0.566	0.501	0.465	0.563	0.638	0.442
		ARGO	0.902	0.551	0.509	0.33	0.435	0.505	0.583	0.383

	Net	0.706	0.623	0.727	0.425	0.548	0.526	0.577	0.509
	ARGONet	–	–	0.53	0.364	0.392	–	–	0.386
MA	GFT	2.409	0.25	0.259	–	–	–	1.148	–
	AR52	0.492	0.239	0.358	0.251	0.279	0.288	0.315	0.264
	ARGO	0.298	0.136	0.173	0.156	0.105	0.174	0.189	0.15
	Net	0.507	0.207	0.296	0.2	0.227	0.269	0.302	0.223
	ARGONet	–	–	0.194	0.185	0.139	–	–	0.164
MD	GFT	1.883	1.381	1.412	–	–	–	1.523	–
	AR52	1.994	0.662	0.95	0.615	0.79	0.987	1.15	0.726
	ARGO	2.031	0.688	0.48	0.615	0.702	0.95	1.111	0.592
	Net	1.989	0.525	0.626	0.597	0.658	0.912	1.066	0.582
	ARGONet	–	–	0.604	0.589	0.664	–	–	0.589
ME	GFT	0.831	0.469	0.662	–	–	–	0.597	–
	AR52	0.551	0.279	0.404	0.351	0.319	0.346	0.366	0.328
	ARGO	0.601	0.27	0.373	0.322	0.239	0.34	0.376	0.295
	Net	0.519	0.293	0.295	0.29	0.229	0.306	0.336	0.255
	ARGONet	–	–	0.32	0.306	0.246	–	–	0.271
MI	GFT	0.504	1.046	0.355	–	–	–	0.682	–
	AR52	0.543	0.341	0.572	0.374	0.445	0.41	0.44	0.41
	ARGO	0.516	0.281	0.515	0.255	0.5	0.388	0.413	0.389
	Net	0.53	0.293	0.465	0.284	0.426	0.371	0.401	0.354
	ARGONet	–	–	0.496	0.284	0.417	–	–	0.364
MN	GFT	3.268	0.578	0.782	–	–	–	1.616	–
	AR52	0.84	0.451	0.941	0.683	0.608	0.637	0.675	0.667
	ARGO	0.847	0.424	0.686	0.629	0.564	0.58	0.598	0.574
	Net	0.857	0.404	0.917	0.6	0.415	0.591	0.659	0.598
	ARGONet	–	–	0.687	0.608	0.464	–	–	0.536
NC	GFT	1.713	0.381	0.47	–	–	–	0.914	–
	AR52	1.181	0.654	1.129	0.522	0.913	0.796	0.878	0.775
	ARGO	0.856	0.435	0.596	0.581	0.738	0.584	0.588	0.56
	Net	1.077	0.406	0.543	0.563	0.727	0.623	0.66	0.536
	ARGONet	–	–	0.556	0.545	0.744	–	–	0.541
ND	GFT	2.609	0.379	1.127	–	–	–	1.61	–
	AR52	1.574	0.703	1.102	0.463	1.266	1.019	1.078	0.953
	ARGO	1.621	0.812	1.365	0.456	1.07	1.068	1.199	0.99
	Net	1.349	0.686	0.886	0.469	1.121	0.891	0.929	0.822
	ARGONet	–	–	1.223	0.48	0.963	–	–	0.898
NE	GFT	2.672	2.397	2.781	–	–	–	2.25	–
	AR52	1.383	0.904	0.801	0.316	0.606	0.844	1.018	0.543
	ARGO	1.239	1.067	0.889	0.398	0.501	0.853	1.036	0.56
	Net	1.111	1.175	1.006	0.516	0.581	0.874	1.043	0.643
	ARGONet	–	–	0.778	0.376	0.501	–	–	0.515
NH	GFT	0.511	0.197	0.299	–	–	–	0.313	–
	AR52	0.46	0.213	0.29	0.201	0.32	0.272	0.295	0.245
	ARGO	0.436	0.168	0.227	0.186	0.278	0.244	0.262	0.214
	Net	0.404	0.152	0.251	0.166	0.272	0.234	0.252	0.213
	ARGONet	–	–	0.247	0.172	0.266	–	–	0.212
	GFT	7.24	0.801	1.585	–	–	–	3.518	–
	AR52	0.649	0.486	0.678	0.545	0.669	0.544	0.545	0.56

NJ	ARGO	0.857	0.432	0.467	0.593	0.707	0.556	0.547	0.524
	Net	0.654	0.446	0.512	0.441	0.703	0.506	0.496	0.502
	ARGONet	–	–	0.5	0.477	0.683	–	–	0.493
NM	GFT	2.683	0.972	1.576	–	–	–	1.624	–
	AR52	0.898	0.587	0.632	0.695	0.395	0.577	0.616	0.528
	ARGO	0.979	0.578	0.511	0.577	0.298	0.554	0.624	0.431
	Net	0.909	0.5	0.583	0.456	0.43	0.522	0.582	0.445
	ARGONet	–	–	0.541	0.462	0.305	–	–	0.405
NV	GFT	1.904	0.789	0.97	–	–	–	1.164	–
	AR52	0.469	0.426	0.484	0.419	0.233	0.358	0.388	0.346
	ARGO	0.374	0.432	0.435	0.321	0.236	0.323	0.354	0.31
	Net	0.532	0.425	0.459	0.391	0.346	0.372	0.396	0.353
	ARGONet	–	–	0.511	0.334	0.23	–	–	0.334
NY	GFT	2.74	0.55	1.158	–	–	–	1.467	–
	AR52	0.675	0.501	0.926	0.687	2.488	1.087	0.61	1.389
	ARGO	0.974	0.413	0.66	1.056	1.925	0.967	0.62	1.171
	Net	0.553	0.455	0.705	0.631	1.998	0.885	0.501	1.124
	ARGONet	–	–	0.591	0.679	1.983	–	–	1.108
OH	GFT	0.854	0.344	0.575	–	–	–	0.526	–
	AR52	0.601	0.249	0.782	0.289	0.398	0.428	0.489	0.46
	ARGO	0.613	0.16	0.51	0.249	0.308	0.345	0.394	0.323
	Net	0.634	0.175	0.56	0.188	0.252	0.349	0.415	0.327
	ARGONet	–	–	0.486	0.204	0.309	–	–	0.306
OR	GFT	1.758	0.538	0.278	–	–	–	0.932	–
	AR52	0.709	0.752	0.372	0.648	0.901	0.62	0.553	0.618
	ARGO	0.697	0.564	0.54	0.592	0.872	0.589	0.526	0.626
	Net	0.56	0.717	0.461	0.599	0.871	0.583	0.514	0.607
	ARGONet	–	–	0.503	0.592	0.932	–	–	0.639
PA	GFT	1.377	0.35	1.003	–	–	–	0.841	–
	AR52	0.632	0.325	0.806	0.443	0.619	0.504	0.52	0.562
	ARGO	0.688	0.309	0.56	0.36	0.433	0.423	0.459	0.406
	Net	0.493	0.239	0.439	0.324	0.55	0.369	0.352	0.396
	ARGONet	–	–	0.463	0.332	0.467	–	–	0.372
RI	GFT	3.446	0.36	0.553	–	–	–	1.745	–
	AR52	0.527	0.288	0.577	0.321	0.102	0.387	0.425	0.398
	ARGO	0.344	0.179	0.296	0.165	0.069	0.248	0.254	0.247
	Net	0.505	0.386	0.369	0.234	0.172	0.341	0.374	0.29
	ARGONet	–	–	0.342	0.158	0.134	–	–	0.259
SC	GFT	2.505	0.733	0.803	–	–	–	1.316	–
	AR52	0.53	0.376	0.798	0.693	1.126	0.639	0.502	0.774
	ARGO	0.348	0.277	0.354	0.749	1.084	0.547	0.283	0.682
	Net	0.506	0.36	0.491	0.579	0.949	0.52	0.387	0.608
	ARGONet	–	–	0.36	0.672	0.977	–	–	0.619
SD	GFT	2.575	0.561	1.097	–	–	–	1.362	–
	AR52	0.252	0.297	0.357	0.308	0.489	0.312	0.276	0.349
	ARGO	0.319	0.254	0.333	0.344	0.366	0.293	0.278	0.314
	Net	0.301	0.326	0.303	0.313	0.438	0.303	0.277	0.319
	ARGONet	–	–	0.305	0.33	0.32	–	–	0.287
	GFT	3.414	0.552	1.053	–	–	–	1.708	–

	TN	AR52	1.065	0.853	0.742	0.937	1.124	0.804	0.738	0.824
		ARGO	0.779	0.385	0.764	0.765	0.93	0.643	0.558	0.73
		Net	1.067	0.52	0.505	0.606	0.859	0.642	0.615	0.614
		ARGONet	–	–	0.663	0.531	0.847	–	–	0.625
	TX	GFT	2.526	1.318	1.8	–	–	–	1.636	–
		AR52	0.836	1.003	1.529	1.097	0.892	0.937	0.977	1.046
		ARGO	0.759	0.942	1.438	0.759	0.73	0.837	0.929	0.905
		Net	0.733	0.964	1.68	0.711	0.806	0.892	1.006	1
		ARGONet	–	–	1.44	0.657	0.75	–	–	0.885
	UT	GFT	1.102	0.657	0.675	–	–	–	0.729	–
		AR52	0.772	0.693	0.531	0.46	0.434	0.529	0.593	0.427
		ARGO	0.819	0.365	0.519	0.537	0.593	0.535	0.546	0.502
		Net	0.676	0.642	0.482	0.489	0.52	0.507	0.538	0.444
		ARGONet	–	–	0.485	0.47	0.466	–	–	0.43
	VA	GFT	1.328	0.436	1.492	–	–	–	0.976	–
		AR52	0.739	0.286	0.901	0.391	0.69	0.539	0.572	0.597
		ARGO	0.588	0.193	0.459	0.175	0.46	0.355	0.382	0.354
		Net	0.662	0.217	0.563	0.191	0.441	0.385	0.431	0.372
		ARGONet	–	–	0.459	0.155	0.428	–	–	0.328
	VT	GFT	1.793	0.986	0.981	–	–	–	1.147	–
		AR52	0.811	0.77	0.841	0.587	0.296	0.63	0.722	0.575
		ARGO	0.646	0.704	0.903	0.589	0.297	0.614	0.697	0.613
		Net	0.698	0.647	0.753	0.436	0.347	0.559	0.644	0.504
		ARGONet	–	–	0.919	0.419	0.318	–	–	0.563
	WA	GFT	2.903	0.523	0.321	–	–	–	1.405	–
		AR52	0.407	0.341	0.389	0.392	0.485	0.345	0.321	0.368
		ARGO	0.405	0.261	0.329	0.335	0.455	0.311	0.286	0.33
		Net	0.413	0.306	0.303	0.386	0.596	0.352	0.294	0.384
		ARGONet	–	–	0.276	0.339	0.519	–	–	0.339
	WI	GFT	2.98	0.958	1.21	–	–	–	1.586	–
		AR52	0.532	0.499	0.801	0.383	0.428	0.506	0.545	0.537
		ARGO	0.45	0.587	0.796	0.403	0.451	0.505	0.548	0.532
		Net	0.425	0.55	0.745	0.376	0.421	0.475	0.51	0.504
		ARGONet	–	–	0.794	0.38	0.402	–	–	0.521
	WV	GFT	1.959	0.474	1.045	–	–	–	1.076	–
		AR52	0.625	0.454	0.777	0.536	–	0.514	0.521	0.593
		ARGO	0.67	0.359	0.457	0.459	–	0.421	0.422	0.407
		Net	0.485	0.334	0.672	0.395	–	0.416	0.429	0.493
		ARGONet	–	–	0.514	0.43	–	–	–	0.423
PEARSON	AK	GFT	0.673	0.932	0.768	–	–	–	0.669	–
		AR52	0.655	0.77	0.772	0.701	0.717	0.751	0.749	0.734
		ARGO	0.695	0.832	0.728	0.804	0.771	0.79	0.765	0.772
		Net	0.667	0.764	0.628	0.818	0.784	0.762	0.713	0.77
		ARGONet	–	–	0.723	0.815	0.75	–	–	0.781
	AL	GFT	0.915	0.867	0.939	–	–	–	0.837	–
		AR52	0.893	0.94	0.792	0.701	0.956	0.86	0.878	0.836
		ARGO	0.955	0.961	0.868	0.598	0.96	0.868	0.916	0.851
		Net	0.939	0.96	0.922	0.738	0.97	0.909	0.942	0.897
		ARGONet	–	–	0.924	0.683	0.969	–	–	0.885

AR	GFT	0.913	0.949	0.968	–	–	–	0.719	–
	AR52	0.939	0.905	0.86	0.53	–	0.899	0.92	0.861
	ARGO	0.951	0.934	0.928	0.481	–	0.883	0.903	0.9
	Net	0.94	0.95	0.906	0.875	–	0.931	0.933	0.926
	ARGONet	–	–	0.909	0.875	–	–	–	0.928
AZ	GFT	0.951	0.913	0.913	–	–	–	0.847	–
	AR52	0.89	0.902	0.955	0.91	0.898	0.942	0.933	0.944
	ARGO	0.949	0.946	0.961	0.932	0.896	0.955	0.959	0.949
	Net	0.919	0.946	0.963	0.903	0.887	0.946	0.953	0.939
	ARGONet	–	–	0.96	0.93	0.896	–	–	0.949
DE	GFT	0.938	0.623	0.712	–	–	–	0.766	–
	AR52	0.927	0.702	0.81	0.623	0.692	0.887	0.875	0.795
	ARGO	0.961	0.757	0.827	0.861	0.823	0.916	0.904	0.845
	Net	0.964	0.749	0.895	0.762	0.441	0.903	0.903	0.857
	ARGONet	–	–	0.841	0.84	0.614	–	–	0.843
GA	GFT	0.91	0.956	0.879	–	–	–	0.843	–
	AR52	0.95	0.895	0.815	0.676	0.847	0.913	0.907	0.875
	ARGO	0.971	0.974	0.927	0.864	0.931	0.929	0.938	0.941
	Net	0.968	0.931	0.82	0.762	0.907	0.927	0.917	0.893
	ARGONet	–	–	0.957	0.711	0.936	–	–	0.945
ID	GFT	0.862	0.903	0.956	–	–	–	0.638	–
	AR52	0.812	0.802	0.897	0.368	–	0.862	0.871	0.897
	ARGO	0.851	0.858	0.918	0.407	–	0.888	0.898	0.905
	Net	0.844	0.852	0.924	0.656	–	0.881	0.879	0.934
	ARGONet	–	–	0.931	0.697	–	–	–	0.929
KS	GFT	0.88	0.954	0.957	–	–	–	0.886	–
	AR52	0.918	0.897	0.949	0.588	0.909	0.933	0.946	0.934
	ARGO	0.943	0.94	0.951	0.765	0.962	0.955	0.953	0.958
	Net	0.925	0.932	0.955	0.848	0.927	0.952	0.957	0.955
	ARGONet	–	–	0.96	0.86	0.961	–	–	0.965
KY	GFT	0.902	0.922	0.959	–	–	–	0.856	–
	AR52	0.91	0.908	0.92	0.8	0.896	0.935	0.929	0.931
	ARGO	0.937	0.966	0.946	0.837	0.908	0.941	0.951	0.938
	Net	0.799	0.967	0.924	0.926	0.893	0.936	0.922	0.934
	ARGONet	–	–	0.94	0.923	0.907	–	–	0.941
LA	GFT	0.904	0.947	0.978	–	–	–	0.856	–
	AR52	0.922	0.852	0.94	0.615	0.933	0.937	0.938	0.948
	ARGO	0.967	0.953	0.96	0.825	0.949	0.958	0.959	0.965
	Net	0.96	0.942	0.945	0.703	0.923	0.947	0.953	0.938
	ARGONet	–	–	0.955	0.796	0.958	–	–	0.963
MA	GFT	0.737	0.802	0.931	–	–	–	0.711	–
	AR52	0.825	0.817	0.86	0.868	0.903	0.895	0.885	0.909
	ARGO	0.966	0.97	0.97	0.96	0.99	0.966	0.965	0.975
	Net	0.852	0.864	0.906	0.916	0.934	0.915	0.905	0.937
	ARGONet	–	–	0.966	0.929	0.976	–	–	0.967
MD	GFT	0.58	0.87	0.926	–	–	–	0.693	–
	AR52	0.358	0.567	0.545	0.717	0.836	0.572	0.477	0.714
	ARGO	0.361	0.601	0.901	0.684	0.878	0.63	0.555	0.819
	Net	0.337	0.623	0.83	0.731	0.896	0.663	0.558	0.837

	ARGONet	–	–	0.842	0.718	0.885	–	–	0.824
ME	GFT	0.861	0.77	0.913	–	–	–	0.805	–
	AR52	0.792	0.807	0.814	0.18	0.734	0.807	0.839	0.808
	ARGO	0.807	0.848	0.876	0.328	0.868	0.823	0.844	0.849
	Net	0.819	0.801	0.907	0.454	0.87	0.853	0.867	0.89
	ARGONet	–	–	0.897	0.375	0.854	–	–	0.873
MI	GFT	0.945	0.879	0.976	–	–	–	0.855	–
	AR52	0.902	0.876	0.843	0.805	0.91	0.914	0.901	0.914
	ARGO	0.917	0.929	0.933	0.93	0.924	0.925	0.916	0.926
	Net	0.907	0.916	0.923	0.899	0.916	0.931	0.919	0.94
	ARGONet	–	–	0.919	0.908	0.928	–	–	0.936
MN	GFT	0.783	0.818	0.917	–	–	–	0.701	–
	AR52	0.817	0.864	0.827	0.618	0.867	0.853	0.855	0.854
	ARGO	0.862	0.88	0.912	0.721	0.883	0.884	0.891	0.896
	Net	0.848	0.889	0.832	0.7	0.935	0.878	0.869	0.884
	ARGONet	–	–	0.91	0.703	0.915	–	–	0.907
NC	GFT	0.873	0.94	0.972	–	–	–	0.878	–
	AR52	0.805	0.75	0.834	0.832	0.878	0.85	0.816	0.875
	ARGO	0.895	0.901	0.956	0.887	0.939	0.926	0.924	0.94
	Net	0.837	0.927	0.96	0.879	0.924	0.913	0.905	0.943
	ARGONet	–	–	0.958	0.89	0.919	–	–	0.942
ND	GFT	0.565	0.928	0.812	–	–	–	0.672	–
	AR52	0.701	0.776	0.804	0.675	0.558	0.807	0.828	0.751
	ARGO	0.681	0.92	0.76	0.663	0.718	0.797	0.794	0.772
	Net	0.804	0.805	0.881	0.675	0.729	0.856	0.875	0.834
	ARGONet	–	–	0.808	0.658	0.761	–	–	0.809
NE	GFT	0.917	0.795	0.819	–	–	–	0.764	–
	AR52	0.871	0.41	0.791	0.589	0.796	0.883	0.874	0.828
	ARGO	0.906	0.711	0.809	0.622	0.874	0.881	0.866	0.856
	Net	0.918	0.571	0.823	0.742	0.829	0.884	0.873	0.846
	ARGONet	–	–	0.861	0.68	0.863	–	–	0.878
NH	GFT	0.915	0.775	0.898	–	–	–	0.873	–
	AR52	0.859	0.672	0.846	0.809	0.706	0.843	0.857	0.825
	ARGO	0.886	0.779	0.908	0.864	0.791	0.88	0.898	0.874
	Net	0.865	0.825	0.883	0.91	0.804	0.882	0.889	0.875
	ARGONet	–	–	0.887	0.894	0.813	–	–	0.877
NJ	GFT	0.841	0.829	0.872	–	–	–	0.784	–
	AR52	0.896	0.758	0.767	0.897	0.914	0.915	0.869	0.93
	ARGO	0.938	0.873	0.898	0.934	0.928	0.916	0.911	0.937
	Net	0.928	0.791	0.904	0.931	0.911	0.932	0.917	0.943
	ARGONet	–	–	0.887	0.922	0.922	–	–	0.944
NM	GFT	0.874	0.892	0.944	–	–	–	0.866	–
	AR52	0.823	0.904	0.876	0.809	0.938	0.891	0.889	0.896
	ARGO	0.853	0.908	0.939	0.883	0.967	0.907	0.9	0.936
	Net	0.873	0.946	0.927	0.93	0.939	0.916	0.911	0.931
	ARGONet	–	–	0.923	0.927	0.966	–	–	0.943
NV	GFT	0.73	0.941	0.92	–	–	–	0.798	–
	AR52	0.883	0.883	0.914	0.812	0.876	0.91	0.918	0.909
	ARGO	0.925	0.88	0.932	0.902	0.875	0.928	0.934	0.929

	Net	0.863	0.885	0.921	0.839	0.831	0.907	0.915	0.908
	ARGONet	–	–	0.896	0.881	0.884	–	–	0.915
NY	GFT	0.864	0.875	0.883	–	–	–	0.745	–
	AR52	0.878	0.853	0.861	0.755	0.651	0.798	0.897	0.764
	ARGO	0.876	0.948	0.934	0.74	0.798	0.849	0.913	0.837
	Net	0.922	0.905	0.925	0.791	0.781	0.874	0.939	0.853
	ARGONet	–	–	0.948	0.751	0.782	–	–	0.856
OH	GFT	0.846	0.95	0.97	–	–	–	0.89	–
	AR52	0.887	0.873	0.831	0.764	0.866	0.885	0.885	0.863
	ARGO	0.89	0.961	0.932	0.839	0.931	0.931	0.931	0.935
	Net	0.882	0.955	0.927	0.91	0.958	0.927	0.919	0.936
	ARGONet	–	–	0.941	0.897	0.932	–	–	0.943
OR	GFT	0.85	0.947	0.822	–	–	–	0.83	–
	AR52	0.809	0.801	0.676	0.671	0.734	0.814	0.82	0.801
	ARGO	0.856	0.887	0.854	0.732	0.794	0.851	0.872	0.82
	Net	0.897	0.822	0.793	0.748	0.753	0.839	0.859	0.812
	ARGONet	–	–	0.779	0.75	0.745	–	–	0.802
PA	GFT	0.873	0.954	0.943	–	–	–	0.818	–
	AR52	0.887	0.913	0.869	0.773	0.872	0.905	0.911	0.889
	ARGO	0.931	0.94	0.95	0.89	0.957	0.943	0.947	0.946
	Net	0.942	0.955	0.971	0.908	0.907	0.951	0.962	0.948
	ARGONet	–	–	0.963	0.905	0.935	–	–	0.954
RI	GFT	0.922	0.874	0.937	–	–	–	0.799	–
	AR52	0.877	0.82	0.873	0.832	-0.077	0.879	0.886	0.876
	ARGO	0.971	0.935	0.97	0.959	0.386	0.954	0.963	0.956
	Net	0.902	0.654	0.955	0.914	0.396	0.911	0.917	0.937
	ARGONet	–	–	0.956	0.961	0.047	–	–	0.949
SC	GFT	0.767	0.916	0.975	–	–	–	0.859	–
	AR52	0.882	0.881	0.854	0.543	0.867	0.918	0.892	0.911
	ARGO	0.975	0.945	0.984	0.524	0.896	0.942	0.967	0.938
	Net	0.929	0.911	0.95	0.712	0.926	0.948	0.943	0.951
	ARGONet	–	–	0.983	0.639	0.918	–	–	0.951
SD	GFT	0.787	0.799	0.888	–	–	–	0.775	–
	AR52	0.93	0.841	0.902	0.806	0.894	0.919	0.924	0.91
	ARGO	0.899	0.89	0.916	0.855	0.942	0.931	0.928	0.932
	Net	0.919	0.847	0.927	0.823	0.929	0.932	0.931	0.932
	ARGONet	–	–	0.926	0.805	0.956	–	–	0.941
TN	GFT	0.872	0.96	0.964	–	–	–	0.883	–
	AR52	0.865	0.9	0.928	0.433	0.777	0.896	0.92	0.876
	ARGO	0.932	0.981	0.961	0.563	0.875	0.937	0.96	0.917
	Net	0.876	0.979	0.968	0.823	0.888	0.937	0.945	0.939
	ARGONet	–	–	0.953	0.841	0.89	–	–	0.936
TX	GFT	0.928	0.918	0.918	–	–	–	0.805	–
	AR52	0.931	0.894	0.85	0.684	0.928	0.902	0.909	0.886
	ARGO	0.962	0.923	0.87	0.725	0.958	0.923	0.919	0.914
	Net	0.947	0.904	0.83	0.73	0.948	0.912	0.904	0.896
	ARGONet	–	–	0.87	0.791	0.954	–	–	0.919
	GFT	0.847	0.84	0.896	–	–	–	0.869	–
	AR52	0.888	0.668	0.902	0.867	0.893	0.901	0.893	0.924

	UT	ARGO	0.886	0.907	0.926	0.849	0.836	0.902	0.915	0.891
		Net	0.915	0.748	0.929	0.834	0.84	0.911	0.916	0.916
		ARGONet	–	–	0.93	0.849	0.88	–	–	0.921
	VA	GFT	0.948	0.977	0.973	–	–	–	0.829	–
		AR52	0.918	0.955	0.9	0.785	0.865	0.926	0.934	0.91
		ARGO	0.964	0.986	0.985	0.961	0.945	0.971	0.974	0.971
		Net	0.94	0.978	0.963	0.957	0.946	0.964	0.965	0.967
		ARGONet	–	–	0.983	0.97	0.95	–	–	0.975
	VT	GFT	0.766	0.701	0.818	–	–	–	0.701	–
		AR52	0.864	0.743	0.888	0.668	0.807	0.872	0.853	0.875
		ARGO	0.915	0.826	0.898	0.676	0.826	0.891	0.877	0.897
		Net	0.899	0.798	0.905	0.818	0.741	0.902	0.887	0.908
		ARGONet	–	–	0.845	0.833	0.782	–	–	0.889
	WA	GFT	0.935	0.933	0.959	–	–	–	0.8	–
		AR52	0.884	0.862	0.936	0.734	0.846	0.901	0.924	0.899
		ARGO	0.903	0.928	0.952	0.801	0.871	0.922	0.941	0.919
		Net	0.918	0.926	0.957	0.737	0.758	0.903	0.945	0.888
		ARGONet	–	–	0.967	0.798	0.823	–	–	0.913
	WI	GFT	0.922	0.557	0.91	–	–	–	0.655	–
		AR52	0.893	0.642	0.813	0.618	0.866	0.888	0.899	0.883
		ARGO	0.931	0.66	0.863	0.64	0.868	0.895	0.903	0.894
		Net	0.938	0.693	0.873	0.731	0.876	0.906	0.915	0.901
		ARGONet	–	–	0.868	0.696	0.895	–	–	0.898
	WV	GFT	0.974	0.929	0.96	–	–	–	0.815	–
		AR52	0.907	0.913	0.934	0.626	–	0.932	0.944	0.926
		ARGO	0.95	0.97	0.984	0.837	–	0.958	0.964	0.968
		Net	0.946	0.955	0.966	0.776	–	0.958	0.964	0.958
		ARGONet	–	–	0.979	0.793	–	–	–	0.965
MAPE	AK	GFT	1.213	0.352	0.737	–	–	–	0.94	–
		AR52	0.448	0.409	0.484	0.481	0.349	0.571	0.653	0.462
		ARGO	0.514	0.466	0.579	0.448	0.359	0.596	0.713	0.461
		Net	0.49	0.416	0.71	0.458	0.306	0.581	0.706	0.475
		ARGONet	–	–	0.595	0.475	0.334	–	–	0.465
	AL	GFT	2.503	1.248	0.843	–	–	–	2.084	–
		AR52	0.419	0.164	0.244	0.798	0.514	0.521	0.418	0.547
		ARGO	0.54	0.151	0.226	0.72	0.426	0.492	0.431	0.494
		Net	0.367	0.147	0.204	0.542	0.385	0.41	0.339	0.455
		ARGONet	–	–	0.179	0.705	0.392	–	–	0.486
	AR	GFT	3.467	0.26	0.178	–	–	–	1.378	–
		AR52	0.428	0.262	0.361	0.516	–	0.525	0.51	0.521
		ARGO	0.838	0.331	0.299	0.411	–	0.842	0.891	0.574
		Net	0.603	0.25	0.196	0.283	–	0.469	0.498	0.308
		ARGONet	–	–	0.221	0.282	–	–	–	0.341
	AZ	GFT	0.296	0.208	0.169	–	–	–	0.197	–
		AR52	0.166	0.122	0.117	0.14	0.144	0.163	0.14	0.174
		ARGO	0.146	0.12	0.097	0.159	0.141	0.153	0.121	0.17
		Net	0.146	0.106	0.101	0.172	0.145	0.152	0.119	0.172
		ARGONet	–	–	0.105	0.158	0.141	–	–	0.168
		GFT	1.072	1.068	2.747	–	–	–	1.59	–

DE	AR52	0.486	0.889	2.346	1.26	1.978	1.363	1.193	1.851
	ARGO	0.64	0.893	2.342	0.97	2.128	1.377	1.263	1.802
	Net	0.381	0.886	3.216	1.403	3.496	1.837	1.43	2.694
	ARGONet	–	–	2.399	1.097	2.693	–	–	2.056
GA	GFT	1.911	1.078	0.782	–	–	–	1.889	–
	AR52	0.153	0.208	0.265	0.194	0.187	0.238	0.258	0.213
	ARGO	0.229	0.214	0.327	0.263	0.221	0.456	0.556	0.281
	Net	0.145	0.182	0.246	0.183	0.166	0.32	0.388	0.194
	ARGONet	–	–	0.211	0.196	0.163	–	–	0.19
ID	GFT	2.677	0.843	0.228	–	–	–	2.303	–
	AR52	0.636	0.375	0.218	0.748	–	0.884	0.794	0.742
	ARGO	0.724	0.482	0.2	0.817	–	0.861	0.759	0.752
	Net	0.702	0.519	0.221	0.495	–	0.957	0.878	0.789
	ARGONet	–	–	0.197	0.526	–	–	–	0.787
KS	GFT	0.882	0.292	0.285	–	–	–	0.688	–
	AR52	0.391	0.286	0.189	0.307	0.251	0.421	0.421	0.37
	ARGO	0.344	0.311	0.23	0.252	0.191	0.484	0.502	0.409
	Net	0.34	0.217	0.195	0.165	0.226	0.372	0.416	0.293
	ARGONet	–	–	0.191	0.203	0.205	–	–	0.315
KY	GFT	2.661	0.531	0.854	–	–	–	2.075	–
	AR52	0.515	0.54	0.477	0.378	0.399	1.122	1.103	0.955
	ARGO	0.425	0.37	0.452	0.268	0.321	0.779	0.87	0.783
	Net	0.711	0.537	0.45	0.24	0.362	1.199	1.388	1.003
	ARGONet	–	–	0.461	0.209	0.324	–	–	0.857
LA	GFT	0.667	0.154	0.103	–	–	–	0.252	–
	AR52	0.135	0.118	0.094	0.163	0.109	0.119	0.12	0.114
	ARGO	0.145	0.109	0.108	0.122	0.11	0.136	0.151	0.117
	Net	0.168	0.126	0.115	0.144	0.127	0.141	0.143	0.137
	ARGONet	–	–	0.089	0.134	0.101	–	–	0.114
MA	GFT	0.642	0.157	0.181	–	–	–	0.319	–
	AR52	0.168	0.135	0.137	0.167	0.15	0.166	0.157	0.165
	ARGO	0.123	0.082	0.074	0.123	0.072	0.138	0.125	0.135
	Net	0.18	0.121	0.139	0.149	0.13	0.17	0.164	0.165
	ARGONet	–	–	0.095	0.131	0.088	–	–	0.135
MD	GFT	0.67	1.308	0.94	–	–	–	1.213	–
	AR52	0.523	0.541	0.25	0.204	0.329	0.409	0.498	0.26
	ARGO	0.565	0.547	0.2	0.224	0.349	0.426	0.515	0.266
	Net	0.476	0.388	0.237	0.229	0.253	0.359	0.425	0.244
	ARGONet	–	–	0.222	0.224	0.26	–	–	0.244
ME	GFT	0.306	0.343	0.417	–	–	–	0.401	–
	AR52	0.199	0.235	0.201	0.26	0.214	0.255	0.231	0.273
	ARGO	0.221	0.23	0.212	0.213	0.187	0.249	0.234	0.262
	Net	0.241	0.219	0.181	0.228	0.185	0.247	0.238	0.243
	ARGONet	–	–	0.192	0.217	0.19	–	–	0.246
MI	GFT	0.205	0.52	0.288	–	–	–	0.454	–
	AR52	0.168	0.124	0.426	0.269	0.155	0.288	0.317	0.359
	ARGO	0.19	0.132	0.533	0.202	0.211	0.318	0.365	0.399
	Net	0.184	0.108	0.406	0.205	0.171	0.292	0.326	0.365
	ARGONet	–	–	0.45	0.216	0.155	–	–	0.385

MN	GFT	1.127	0.366	0.29	–	–	–	0.727	–
	AR52	0.301	0.34	0.226	0.434	0.22	0.449	0.504	0.326
	ARGO	0.366	0.318	0.235	0.455	0.23	0.443	0.473	0.357
	Net	0.38	0.252	0.245	0.433	0.161	0.43	0.504	0.296
	ARGONet	–	–	0.232	0.429	0.188	–	–	0.313
NC	GFT	0.904	0.2	0.236	–	–	–	0.534	–
	AR52	0.302	0.285	0.261	0.183	0.242	0.428	0.546	0.273
	ARGO	0.351	0.241	0.214	0.196	0.178	0.353	0.447	0.215
	Net	0.322	0.228	0.202	0.172	0.26	0.35	0.433	0.226
	ARGONet	–	–	0.196	0.194	0.22	–	–	0.222
ND	GFT	0.516	0.996	0.471	–	–	–	0.696	–
	AR52	0.349	1.588	0.647	0.701	0.894	0.811	0.832	0.768
	ARGO	0.345	2.168	0.786	0.656	0.796	0.994	1.098	0.812
	Net	0.326	1.573	0.625	0.624	0.947	0.814	0.81	0.759
	ARGONet	–	–	0.62	0.646	0.783	–	–	0.764
NE	GFT	0.32	2.003	1.574	–	–	–	1.157	–
	AR52	0.218	1.034	0.366	0.518	0.411	0.558	0.606	0.471
	ARGO	0.181	1.316	0.384	0.635	0.44	0.607	0.648	0.492
	Net	0.192	1.234	0.465	0.825	0.487	0.629	0.63	0.572
	ARGONet	–	–	0.385	0.623	0.439	–	–	0.492
NH	GFT	0.624	0.388	0.616	–	–	–	0.618	–
	AR52	0.666	0.685	0.584	0.316	0.338	0.587	0.757	0.432
	ARGO	0.471	0.547	0.826	0.32	0.294	0.539	0.674	0.49
	Net	0.484	0.505	0.691	0.265	0.287	0.484	0.609	0.413
	ARGONet	–	–	0.623	0.282	0.279	–	–	0.422
NJ	GFT	1.035	0.244	0.319	–	–	–	0.508	–
	AR52	0.196	0.145	0.196	0.33	0.13	0.279	0.269	0.258
	ARGO	0.266	0.146	0.155	0.492	0.161	0.301	0.263	0.291
	Net	0.221	0.153	0.141	0.267	0.145	0.256	0.242	0.232
	ARGONet	–	–	0.143	0.28	0.138	–	–	0.222
NM	GFT	0.845	0.31	0.63	–	–	–	0.641	–
	AR52	0.189	0.133	0.164	0.242	0.13	0.195	0.186	0.205
	ARGO	0.244	0.148	0.171	0.202	0.116	0.2	0.21	0.186
	Net	0.268	0.116	0.188	0.188	0.186	0.196	0.192	0.199
	ARGONet	–	–	0.182	0.18	0.122	–	–	0.182
NV	GFT	0.988	0.729	1.253	–	–	–	1.427	–
	AR52	0.237	0.178	0.318	0.3	0.171	0.274	0.287	0.293
	ARGO	0.241	0.209	0.357	0.285	0.181	0.292	0.31	0.319
	Net	0.345	0.172	0.273	0.342	0.262	0.294	0.284	0.323
	ARGONet	–	–	0.316	0.256	0.18	–	–	0.287
NY	GFT	0.689	0.247	0.349	–	–	–	0.495	–
	AR52	0.183	0.176	0.199	0.462	0.49	0.497	0.219	0.725
	ARGO	0.23	0.184	0.21	0.601	0.433	0.574	0.25	0.831
	Net	0.214	0.184	0.187	0.409	0.453	0.409	0.215	0.569
	ARGONet	–	–	0.183	0.433	0.438	–	–	0.569
OH	GFT	0.264	0.358	0.301	–	–	–	0.382	–
	AR52	0.257	0.202	0.213	0.227	0.172	0.269	0.305	0.207
	ARGO	0.301	0.15	0.253	0.183	0.152	0.242	0.284	0.191
	Net	0.274	0.158	0.199	0.164	0.132	0.224	0.262	0.191

	ARGONet	–	–	0.191	0.172	0.14	–	–	0.166
OR	GFT	1.529	1.027	0.511	–	–	–	1.379	–
	AR52	0.584	0.805	0.533	0.525	0.322	0.625	0.677	0.555
	ARGO	0.725	0.739	0.518	0.585	0.357	0.686	0.77	0.6
	Net	0.779	0.669	0.545	0.488	0.311	0.611	0.697	0.483
	ARGONet	–	–	0.57	0.471	0.353	–	–	0.521
PA	GFT	0.378	0.162	0.27	–	–	–	0.28	–
	AR52	0.157	0.139	0.171	0.136	0.188	0.178	0.175	0.182
	ARGO	0.172	0.186	0.145	0.125	0.192	0.183	0.192	0.157
	Net	0.134	0.142	0.115	0.107	0.172	0.152	0.155	0.141
	ARGONet	–	–	0.118	0.112	0.162	–	–	0.131
RI	GFT	1.8	0.556	1.457	–	–	–	1.583	–
	AR52	0.344	0.452	0.542	0.629	0.46	0.731	0.738	0.722
	ARGO	0.313	0.294	0.368	0.31	0.302	0.558	0.644	0.424
	Net	0.455	0.496	0.67	0.457	0.749	0.764	0.862	0.685
	ARGONet	–	–	0.402	0.289	0.544	–	–	0.47
SC	GFT	1.996	0.796	0.643	–	–	–	1.357	–
	AR52	0.299	0.422	0.429	0.406	0.262	0.718	0.726	0.647
	ARGO	0.296	0.273	0.383	0.336	0.266	0.635	0.67	0.563
	Net	0.288	0.309	0.335	0.307	0.215	0.597	0.658	0.476
	ARGONet	–	–	0.328	0.31	0.226	–	–	0.485
SD	GFT	0.867	0.323	0.301	–	–	–	0.722	–
	AR52	0.201	0.19	0.167	0.149	0.192	0.279	0.327	0.198
	ARGO	0.277	0.176	0.148	0.167	0.158	0.279	0.344	0.175
	Net	0.248	0.191	0.154	0.143	0.15	0.275	0.332	0.178
	ARGONet	–	–	0.15	0.152	0.154	–	–	0.17
TN	GFT	1.221	0.552	0.45	–	–	–	1.121	–
	AR52	0.381	0.355	0.241	0.404	0.227	0.759	1.016	0.365
	ARGO	0.425	0.384	0.31	0.339	0.204	0.681	0.91	0.356
	Net	0.373	0.278	0.305	0.232	0.187	0.659	0.903	0.358
	ARGONet	–	–	0.295	0.225	0.183	–	–	0.321
TX	GFT	0.331	0.275	0.243	–	–	–	0.268	–
	AR52	0.122	0.105	0.181	0.179	0.167	0.153	0.145	0.176
	ARGO	0.113	0.138	0.211	0.139	0.175	0.161	0.168	0.179
	Net	0.14	0.117	0.163	0.141	0.189	0.148	0.144	0.16
	ARGONet	–	–	0.169	0.122	0.185	–	–	0.156
UT	GFT	0.18	0.221	0.17	–	–	–	0.393	–
	AR52	0.147	0.189	0.122	0.189	0.286	0.294	0.328	0.211
	ARGO	0.149	0.141	0.151	0.184	0.492	0.346	0.349	0.296
	Net	0.134	0.187	0.136	0.176	0.328	0.292	0.32	0.219
	ARGONet	–	–	0.134	0.161	0.326	–	–	0.222
VA	GFT	0.341	0.183	0.34	–	–	–	0.256	–
	AR52	0.139	0.082	0.112	0.117	0.143	0.115	0.11	0.121
	ARGO	0.097	0.078	0.127	0.055	0.136	0.114	0.119	0.13
	Net	0.131	0.071	0.139	0.073	0.099	0.105	0.118	0.103
	ARGONet	–	–	0.125	0.054	0.096	–	–	0.097
VT	GFT	0.387	0.242	0.333	–	–	–	0.487	–
	AR52	0.211	0.22	0.268	0.438	0.295	0.341	0.322	0.431
	ARGO	0.184	0.195	0.313	0.438	0.372	0.368	0.35	0.491

	Net	0.198	0.175	0.242	0.428	0.354	0.322	0.273	0.416
	ARGONet	–	–	0.26	0.365	0.359	–	–	0.397
WA	GFT	1.442	0.311	0.243	–	–	–	0.672	–
	AR52	0.214	0.28	0.238	0.552	0.278	0.353	0.289	0.389
	ARGO	0.272	0.285	0.2	0.337	0.417	0.34	0.289	0.353
	Net	0.239	0.254	0.185	0.481	0.48	0.334	0.267	0.361
	ARGONet	–	–	0.177	0.36	0.36	–	–	0.309
WI	GFT	1.462	0.493	0.467	–	–	–	0.715	–
	AR52	0.293	0.232	0.221	0.177	0.251	0.5	0.468	0.493
	ARGO	0.428	0.258	0.246	0.18	0.264	0.509	0.525	0.464
	Net	0.263	0.261	0.26	0.155	0.224	0.446	0.455	0.424
	ARGONet	–	–	0.256	0.167	0.216	–	–	0.437
WV	GFT	1.279	0.249	0.307	–	–	–	0.674	–
	AR52	0.263	0.255	0.205	0.249	–	0.27	0.281	0.23
	ARGO	0.287	0.222	0.182	0.2	–	0.261	0.281	0.197
	Net	0.284	0.212	0.224	0.179	–	0.272	0.299	0.206
	ARGONet	–	–	0.188	0.19	–	–	–	0.202
